# Potential Target miR-455 Delaying Arterial Stenosis Progression Through PTEN

**DOI:** 10.3389/fcvm.2021.611116

**Published:** 2021-02-23

**Authors:** Ruoran Lin, Junyuan Lv, Lei Wang, Xuan Li, Jing Zhang, Weifeng Sun, Xiaoyun Hu, Shijie Xin

**Affiliations:** ^1^Department of Vascular Surgery, The First Hospital of China Medical University, Shenyang, China; ^2^Department of Breast and Thyroid Surgery, Affiliated Hospital of Zunyi Medical University, Zunyi, China; ^3^Liaoning Key Laboratory of Molecular Tumor Drug Development and Evaluation, Department of Pharmacology, School of Pharmacy, China Medical University, Shenyang, China; ^4^Department of Gastrointestinal Surgery, The First Affiliated Hospital of Dalian Medical University, Dalian, China

**Keywords:** vascular smooth muscle cell, stenosis, phenotype, miR-455, proliferation

## Abstract

**Background:** Vascular smooth muscle cells (VSMC) underwent phenotypic switching upon stimulation signals, and this is the prerequisite for their proliferation and migration. Previous work revealed that miR-455 may be involved in vascular stenosis. Thus, this study aimed to explore potential targets and mechanisms underlying the dynamics of miR-455 in vascular stenosis.

**Methods:** miR-455 and PTEN expression levels were studied in normal and stenosis tissue, as well as in VSMC in proliferation model. Manipulating miR-455 expression levels was achieved by transfection of either miR-455 mimic or inhibitor, and its effect on cell proliferation was studied by CCK-8 assay. Its effect on gene expression was studied by RT-qPCR and western blot. The expression regulation mechanism was studied by luciferase reporter system. Finally, the effect of miR-455 on regulating vascular stenosis was studied using a rat balloon-injured carotid artery stenosis model.

**Results:** High expression levels of miR-455 were detected in both stenosis arterial tissues and VSMC proliferation models. In contrast, the expression levels of PTEN were downregulated in these systems. miR-455 transfected VSMC showed higher levels of proliferation and decreased levels of PTEN. Potential binding sites between miR-455 and PTEN 3′UTR were predicted and confirmed. NF-kB p65 was found to bind directly on miR-455 promoter region and regulate its transcription. The progression of arterial stenosis could be delayed by introducing miR-455 antagomir.

**Conclusions:** The p65/miR-455/PTEN signaling pathway plays a crucial role in regulating VSMC proliferation and vascular stenosis. This indicated that miR-455 is a novel target that would help improve treatment outcomes in patients suffering from vascular stenosis.

## Introduction

Cardiovascular disease is one of the major causes of morbidity and mortality in many countries, and its main pathological basis is atherosclerosis. Arteriosclerosis obliterans (ASO) is a type of most commonly seen peripheral vascular diseases, in which the patients experience ischemia in their lower limbs. There is currently no conclusive treatment for ASO. Surgery may be effective for reconstruction of blood flow. However, the efficacy is not satisfactory. Within 1-year post-surgery, 30–50% of patients develop restenosis and 12% suffer from severe ischemia and eventually experience amputation (1, 2). The mechanism of atherosclerosis is not entirely clear. It is believed that aberrant proliferation and migration of vascular smooth muscle cells (VSMCs) are key aspects of development of atherosclerosis and postoperative restenosis. VSMCs maintain a contractile phenotype within mature blood vessels, hallmarked by the expression of a series of smooth muscle cell-specific contractile markers, including SMA-α. During injury response, VSMCs underwent phenotypic switch, in which the contractile marker expressions decrease, and synthetic marker increases. VSMCs become capable of proliferating and migrating. This phenotypic switch is regulated by a number of signaling pathways that have not been fully understood.

MicroRNAs (miRs) are noncoding RNAs (ncRNAs) composed of 21–25 nucleotides (3, 4). miRs usually regulate their target gene expression through binding to the 3′ untranslated region (3′UTR) of mRNAs (5). A number of studies have demonstrated that miRs are involved in cardiovascular disease progression (2, 6, 7). For example, H19 derived miR-675 levels increased in neointimas of balloon-injured rat carotid arteries, and it's shown to target PTEN 3′UTR in regulating vascular restenosis (8). miR-126 preserves endothelial function while preventing VSMC proliferation during restenosis progression (9). There is also evidence indicating that ablation of inflammatory miR-21 reduces neointimal formation after stent in mice (10). miR-352 is identified as one of the miRNAs down-regulated during fluid shear stressed collaterals, and it may negatively regulate arteriogenesis (11).

miR-455 was identified as one of the tumor suppressor miRs in a number of studies (12–14), although conflicting results also exist (15, 16). Several studies showed that miR455 is part of the inflammatory miRs, although its specific role in inflammation is not definitive (17–20). Studies have shown that miR-455 inhibits pulmonary arterial hypertension through FGF7 modulation (21), and that its expression levels are increased in a rat carotid artery injury model (22). There's also evidence that NF-kB may be responsible in regulating miR-455 levels (19).

The classic tumor suppressor PTEN has been shown as a key regulator in VSMC phenotypic switching. It was detected in early vascular injury, especially upregulated in apoptotic VSMCs (23). And its repression is related to vascular remodeling (24, 25). In addition, the interaction between NF-κB p65 and PTEN influenced VSMC proliferation, de-differentiation and cytokine production during remodeling (26).

In this present study, we set to investigate the role of miR-455 in regulating the phenotypic switching of VSMC. We decided to use bioinformatics and cellular biology approaches, as well as animal model to study the regulatory relationship between miR-455 and PTEN, and the role of NF-κB p65 as an upstream regulator in stenosis. We aimed to assess whether p65/miR-455/PTEN pathway could be a candidate for therapeutic target for stenosis.

## Materials and Methods

### Rat Carotid Artery Balloon Injury Model and *in vivo* Transfections

Male Sprague-Dawley (SD) rats weighing between 300 and 400 g were used in establishing carotid artery balloon injury model. Animal protocols were approved by the Institutional Animal Care and Use Committee at the Chinese Medical University and were consistent with the Guide for the Care and Use of Laboratory Animals ([2019]2019-120-2). The procedure was carried out as described in Tulis (27). Five rats underwent the procedure as model group, while the other five did not receive injury as sham group. In the experiment that *in vivo* transfections were performed, 15 balloon injured rats were divided into three groups at five/group, each group received transfection by mixing rno-miR-455 agomiR, antagomir, or NC with Matrigel, and smearing the mixture around the left external carotid artery. The miRNA agomir and antagomir used in animal transfection experiment were obtained from RiboBio (Guangzhou, China). micrON™ miRNA agomir is a chemically modified double strand miRNA mimics that can upregulate the endogenous miRNA activity by using the cellular machinery. It is stable for *in vivo* study. micrOFF™ miRNA antagomir is a synthetic RNA that can sequence specifically bind to endogenous mature miRNA to prevent its binding to its target sequence. Due to its chemical modification, it has better stability than other inhibitors *in vivo*. Finally, 4% paraformaldehyde was used to fix specimens before paraffin embedding.

### Human Artery Samples

Sections of healthy arteries (renal artery) from organ donors (five cases) that were otherwise discarded during kidney transplantation were obtained after surgery. Stenotic arteries from ASO patients (five cases) were obtained from the lower limb that was amputated. All procedures were approved by the Ethics Committee at the Chinese Medical University, The First Affiliated Hospital of China Medical University and informed consent was obtained from all enrolled patients. Samples were removed of Intima and externa, and washed repletely with DEPC water before RNA extraction.

### Tissue Analysis

After 24 h of fixation in 4% paraformaldehyde and processing in 70% ethanol, tissues were embedded in paraffin. Paraffin sections with 5-μm thickness were stained with Hematoxylin and Eosin (H&E). Each section was examined for four random and noncontiguous microscopic fields to facilitate calculations of mean thickness of intima and media. Then, intimal and medial area were calculated, and the intimal to medial (I/M) area ratio of each section were computed using Image-Pro Plus 6.0 Software (Media Cybernetics, USA).

### Cell Culture and Establishment of the Cell Proliferation Model

The human aortic vascular smooth muscle cell line T/G HA-VSMC (ATCC CEL-1999) or human embryonic kidney 293T cells (ATCC CRL-3216) were maintained and cultured in Roswell Park Memorial Institute (RPMI) 1640 Medium or Dulbecco's Modified Eagle's medium supplemented with 10% FBS (PAN Biotech, Germany) and 1% penicillin/G- streptomycin sulfate in a 5% CO_2_ humidified atmosphere with a constant temperature of 37°C.

The T/G HA-VSMCs proliferation model was established by culturing cells in RPMI 1640 Medium containing 20% FBS and 1% penicillin/G- streptomycin sulfate in a 5% CO_2_ humidified atmosphere with a constant temperature of 37°C (28).

### Transfections With microRNAs

A monolayer of T/G HA-VSMCs was plated in 96-well plates at a density of 3 × 10^4^ cells/ml. When cells reached a confluency of 70%, they were transfected with microRNA oligonucleotides. The miRNA mimic and inhibitor used in HA-VSMC transfection experiments were obtained from RiboBio (Guangzhou, China). micrON™ miRNA mimic is a chemically synthesized product that can mimic high expression of mature miRNA in cells. micrOFF™ miRNA inhibitor is a chemically modified sequence specific miRNA inhibitor that binds to mature miRNA in cells to reduce its function. In addition, Lipofectamine 2000 was used for transfections based on protocols provided by Invitrogen.

### Cell-Counting Kit-8 (CCK-8) Assay

CCK-8 (Beyotime Biotechnology, China) kit was used to assess cell proliferation. Cells were seeded in 96-well plates (density of 5 × 10^3^ cells/well) and cultured in Roswell Park Memorial Institute (RPMI) 1640 Medium containing 10% FBS for 24 h. A total of 10 μL of CCK-8 reagent was added to each well and cells were incubated at 37°C for 0.5–4 h. Absorbance was measured (optical density, OD) at a wavelength of 450-nm using an enzyme immunoassay analyzer (Bio-Rad, USA). All experiments were done in triplicate.

### Cell Cycle Assays

Cell cycle was analyzed by flow cytometry. Briefly, T/G HA-VSMCs were cultured in 6-well plates under various conditions for about 24 h before they were fixed in 70% cold ethanol at 4°C overnight. Cells were washed two times with ice-cold PBS, followed by propidium iodide (PI) staining with buffer containing 10 mg/mL RNase at 37°C; cells were analyzed with flow cytometry. Flow cytometry results were analyzed using FlowJo software and the percentage of cells in the S phase of the cell cycle was determined. All experiments were done in triplicate.

### Immunoblotting

Protein was extracted from cells using RIPA buffer containing PMSF (Beyotime Biotechnology, China). Total protein levels were quantified using the BCA protein assay (CWBio, China). Proteins were resolved by sodium dodecyl sulfate-polyacrylamide gel electrophoresis and transferred to polyvinylidene fluoride membranes. Membranes were blocked in 1% BSA before the incubation with various primary antibodies (Table 1) overnight at 4°C. Secondary antibody incubations were carried out at room temperature for 1 h. Immune complexes were detected and evaluated using the SuperSignal West Femto Maximum Sensitivity Substrate (Thermo Fisher Scientific, USA) and the band density was quantified with Image J (NIH, USA).

**Table 1:** Antibodies used.

**Antibody**	**Manufacture**	**Catalog number**
NF-kb/p65	Cell Signaling Technology	#6956
PTEN	BOSTER	bm4114
Alpha-SMA	Abcam	ab5694
OPN	Abcam	ab8448
Beta-actin	BOSTER	bm3873

### Luciferase Reporter Assay

3′UTR of PTEN containing predicted binding targets of miR-455 was cloned into the luciferase reporter plasmid (Ribobio Co., Ltd., China) with either original sequence (WT) or altered sequence at the binding sites (MT). 293T cells were used for luciferase reporter assay. 293T cells were seeded in 96-well plates at a density of 8 × 10^3^ cells per well for 24 h before co-transfection with WT or MT 3′UTR dual luciferase reporter plasmid vector and miR-455 mimics or miR-NC using Lipofectamine™ 2000 (Invitrogen, USA). Approximately 24 h post-transfection, luciferase assays were performed using the Dual-Luciferase Reporter Assay System (Promega, USA) following protocols provided by the manufacturer. Luciferase activities were represented as the ratio of firefly luciferase signal to renilla luciferase signal. An analogous procedure was performed to examine binding effects between the p65 and miR-455 promoter regions. Plasmid containing p65 cDNA was purchased from Addgene (Addgene, USA). All experiments were done in triplicate.

### Fluorescence Microscopy

T/G HA-VSMCs grown on glass coverslips were fixed using 3.7% paraformaldehyde and permeabilized with 0.01% TritonX100. Nonspecific binding was blocked in 5% bovine serum albumin for 1 h. Cells were incubated with a PTEN antibody (Abcam, US) overnight at 4°C and then incubated at room temperature with a secondary antibody (Beyotime Biotechnology, China). DAPI (Beyotime Biotechnology, China) was used for nuclei staining. Immunofluorescence was observed using a fluorescent microscope (Olympus, Japan).

### Reverse Transcription Quantitative Real-Time Polymerase Chain Reaction (RT-qPCR)

RT-qPCR was used to detect the gene expression levels of miR-455, p65, PTEN, OPN and SMA-α. RNA was isolated using the TRIzol Reagent (CWBio, China) or miRNA Purification Kit (CWBio, China). Samples were quantified using a Nanodrop1000 spectrophotometer (Thermo Scientific, USA). To detect miR-455 levels, RNA was reverse transcribed using the M-MLV reverse transcriptase (Takara, Japan) and specific reverse transcription primer (RiboBio, China). To detect p65, PTEN, OPN and SMA-α, RNA was reverse transcribed using the reverse transcription kit (Takara, Japan). β-actin was used as an internal control for mRNA related experiments. PCR primers were listed in Table 2. RT-qPCR was performed using SYBR Green dye on the Biosystems 7500 Real-time PCR system (Applied Biosystems, USA). All primer sets for miR-455 used in this study were produced by RiboBio. Manufacturer protocols provided by PrimeScript™ RT-PCR Kit and the TB Green^®^ *Premix Ex Taq*™ II (Tli RNaseH Plus) (TAKARA, Japan) were used.

**Table 2:** Primer sets.

**Gene**	**Forward primer**	**Reverse primer**
p65	5′-TGTGAAGAAGCGGGACCTGGAG-3′	5′-AAGCAGAGCCGCACAGCATTC-3′
PTEN	5′-GACCAGAGACAAAAAGGGAGTA-3′	5′-ACAAACTGAGGATTGCAAGTTC-3′
OPN	5′-TGATGGCCGAGGTGATAGTG-3'	5′-TCCATGTGTGAGGTGATGTCC-3′
α-SMA	5′-AATGGCGTGATTCTGAGCAA-3′	5′-AACTGAGCCACCTGCTCCAT-3′
β-acting	5′-CATGTACGTTGCTATCCAGGC-3′	5′-CTCCTTAATGTCACGCACGAT-3′

### Chromatin Immunoprecipitation Assay (ChIP)

Chromatin immunoprecipitation (ChIP) was performed using a ChIP Assay Kit (Beyotime Biotechnology, China). Immunoprecipitation was performed with an anti-p65 antibody (Cell Signaling Technology, USA). The final purified DNA fragment was subjected to PCR analysis using Hot-Start Taq DNA polymerase (Takara, Japan). PCR products were analyzed using gel electrophoresis. Sequences for miR-455 promoter primers are as follows: Forward: CGGTGGCTCGTGCCTGTAATC, Reverse: CAAGCGATTCTCCTGCCTCAGTC.

### Statistical Analysis

All results were presented as the mean ± standard deviation. The Kolmogorov-Smirnov was used to assess whether the data fit the normal distribution. When the data didn't fit the normal distribution, Mann-Whiney U tests were used to evaluate comparison among groups. When the data fit normal distribution, unpaired *t*-tests were used. Student's *t*-tests were used to compare differences between two groups. For comparing multiple groups, differences were assessed using one-way ANOVA. Significance was established when the *P*-value was <0.050. SPSS for Windows (SPSS Version 19.0 Inc., USA) was used to analyze data.

## Results

### miR-455 Expression Is Associated With Vascular Restenosis and HA-VSMC Proliferation

**Figure 1:**
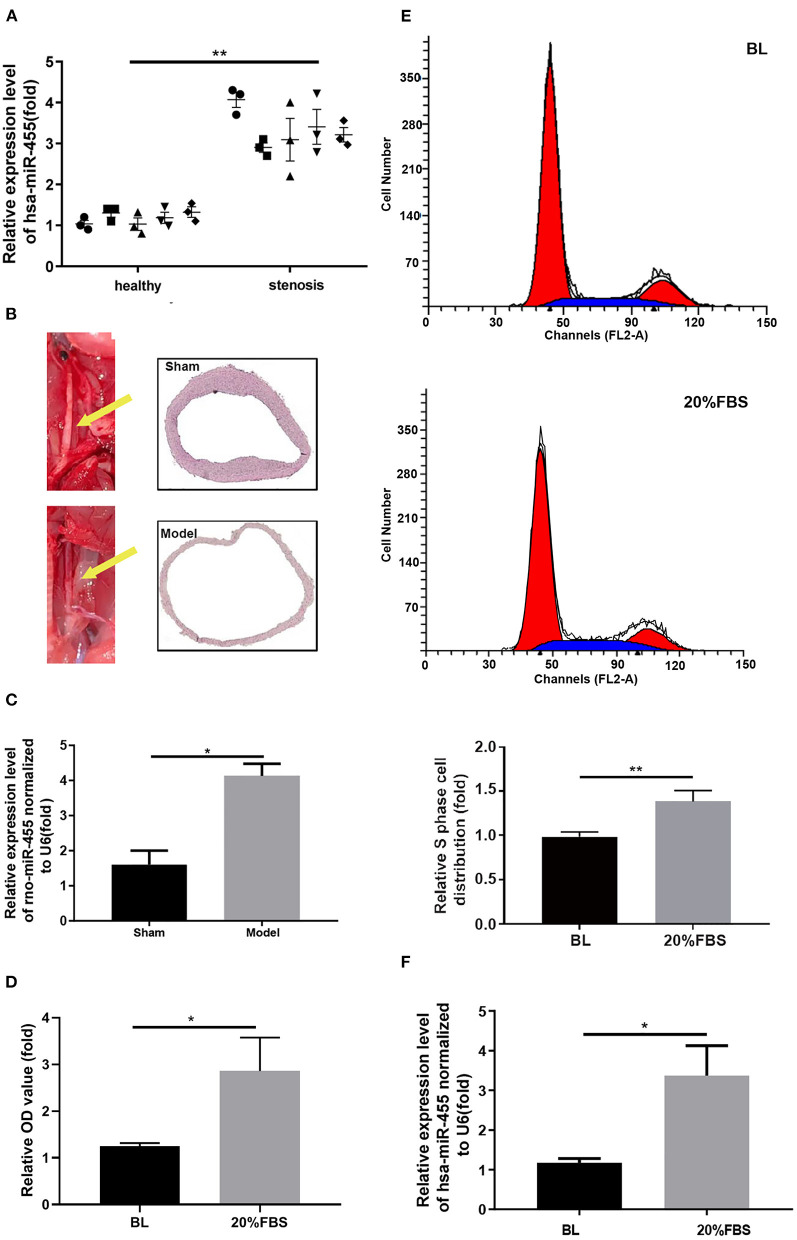
**(A)** Expression levels of hsa-miR-455 in healthy (5) and stenotic (5) arteries examined by qRT-PCR. Hsa-miR-455 was normalized to U6. The first PCR value of the first normal sample was designated as 1. **(B)** The rat carotid artery balloon injury model. The left carotid artery (yellow arrow) was injured. **(C)** Expression levels of rno-miR-455 in balloon-injured and healthy carotid arteries examined by qRT-PCR. Rno-miR-455 was normalized to U6. The PCR value of tissue of the first animal from sham group was designated as 1. **(D)** Proliferation of T/G HA-VSMCs grown before and after 20% FBS stimulation measured by CCK-8 assay. Three biological replicated experiments were performed. **(E)** Representative flow cytometry of cell cycle analysis for T/G HA-VSMCs grown before and after 20% FBS stimulation (Blue region stands for S phase), and quantification of S phase cell percentage. Three biological replicated experiments were performed **(F)** Expression levels of hsa-miR-455 in cells grown before and after 20% FBS examined by qRT-PCR. Hsa-miR-455 level was normalized to U6. Three biological replicated experiments were performed ^*^p < 0.05, ^**^p < 0.01. In all the experiments of **(D–F)**, the values obtained from first biological replica of the base line group were designated as 1.

The expression levels of miR-455 in healthy arteries and stenotic arteries from ASO patients were assessed. The results indicated that has-miR-455 was expressed at lower levels in healthy arteries than it was in the stenotic arteries (Figure 1A).

Similar findings were observed in a rat carotid artery balloon injury model (Figure 1B), where the elevated levels of rno-miR-455 were detected in the balloon-injured carotid arteries comparing to those in the Sham group (Figure 1C).

T/G HA-VSMC was chosen for establishing *in vitro* proliferation model by growing the cells in 20% FBS. T/G HA-VSMC is a human VSMC derived from normal aorta. It expresses VSMC contractile markers and is a good tool for studying the VSMC phenotypic switching without the constrains of availability of primary cells. Cell proliferation was assessed by CCK-8 assay (Figure 1D). Propidium iodide staining showed that more cells entered into S phase when cells were grown in 20% FBS (Figure 1E). Increased levels of hsa-miR-455 were detected in the T/G HA-VSMC grown in 20% FBS comparing to the cells grown in normal growth condition (Figure 1F). These results indicated that miR-455 levels were correlated closely with vascular stenosis and excessive proliferation of T/G HA-VSMCs.

### miR-455 Induce VSMC Proliferation, Phenotypic Switching

**Figure 2:**
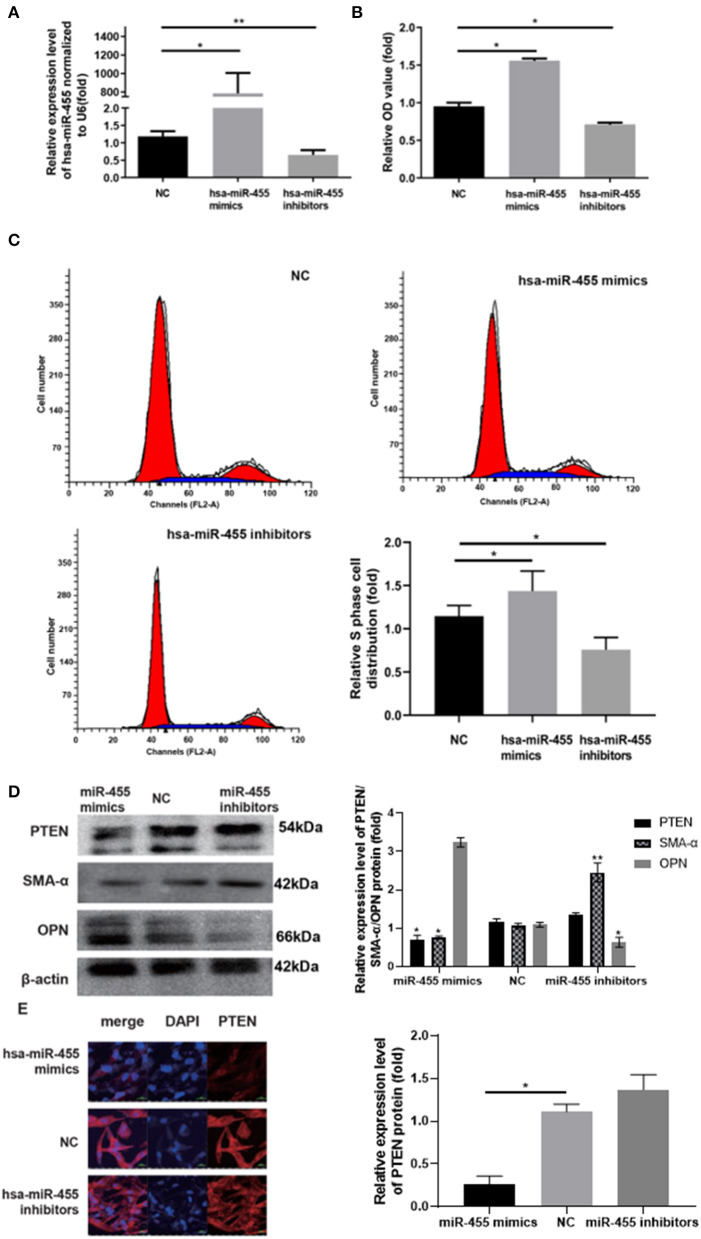
**(A)** Expression levels of hsa-miR-455 in T/G HA-VSMCs transfected with hsa-miR-455 mimics, inhibitors, or NC examined by qRT-PCR. Hsa-miR-455 was normalized to U6. **(B)** Proliferation of T/G HA-VSMCs after transfecting hsa-miR-455 mimics, inhibitors, or NC measured by CCK-8 assay. **(C)** Representative flow cytometry of cell cycle analysis after transfecting hsa-miR-455 mimics, inhibitors or NC (Blue region stands for S phase), and quantification of S phase percentage. **(D)** Representative images of immunoblotting for PTEN, SMA-α, and OPN expression levels in T/G HA-VSMCs transfected with hsa-miR-455 mimics, inhibitors, or NC, and average band density of three replicates normalized against β-actin. **(E)** Representative images from fluorescent microscopy for the different groups. ^*^p < 0.05, ^**^p < 0.01. All experiments were performed with three biological replicates. In all experiments, the values obtained from the first biological replica of negative control transfection were designated as 1.

To investigate whether miR-455 is responsible for the proliferation of VSMCs and phenotypic switching, hsa-miR-455 mimics or hsa-miR-455 inhibitors were transfected into T/G HA-VSMC cells, the proliferation of the transfected cells were compared with the negative control (NC). RT-qPCR demonstrated that miR-455 mimics or inhibitors were effective in inducing overexpression or repressing the expression of miR-455, respectively (Figure 2A). Cell proliferations were assessed by CCK-8 assay and demonstrated that overexpression of miR-455 increased cell proliferation comparing to the NC, while repression of miR-455 reduced cell proliferation (Figure 2B). The increased cell proliferation with miR-455 mimic transfection was confirmed by the increased proportion of cells that were in S phase as analyzed with flow cytometry (Figure 2C). On the other hand, cells transfection with a hsa-miR-455 inhibitor showed a decreased the distribution of S phase cells.

The expression levels of contractile marker SMA-α and synthetic marker osteopontin (OPN), were analyzed with western blot (Figure 2D). It was found that SMA-α levels were decreased in has-miR-455 mimic transfected cells comparing to negative control (NC), but increased in has-miR-455 inhibitor transfected cells; while the OPN level changes showed the opposite pattern. These findings suggested miR-455 was able to induce phenotypic switching in VSMC.

Study has suggested that miR-455 may target PTEN in asserting its effect (29). Expression levels of PTEN were analyzed with western blot and immunofluorescence in has-miR-455 mimic or inhibitor transfected cells. Western blot study showed that PTEN expression levels decreased in has-miR-455 mimic transfected T/G HA-VSMCs, while they increased in hsa-miR-455 inhibitor transfected cells (Figure 2D). Similar results were seen with immunofluorescence staining on VSMC (Figure 2E).

Note that while using the same concentration of miR-455 mimic and inhibitor in transfection experiments, the mimic clearly had notably higher transfection efficiency which led to almost 800-folds of increase comparing to NC (Figure 2A). Although this may not be seen under physiological condition, due to the limited availability of other necessary cellular machineries, such as AGO proteins, the excess amount of miR-455 may not be able to assert its function in such an excess capacity.

### miR-455 Directly Targets PTEN

**Figure 3:**
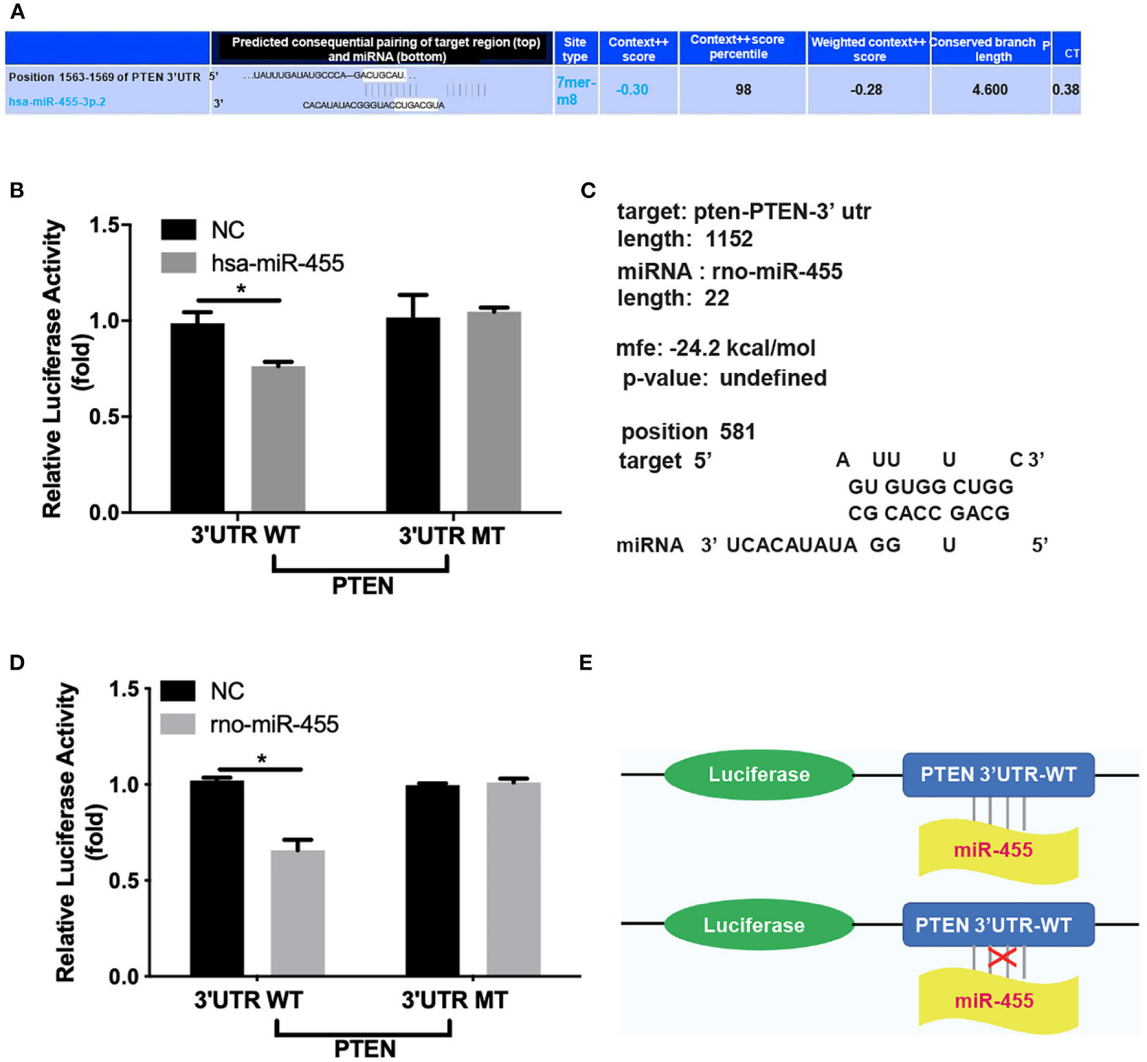
**(A)** Potential binding sites between hsa-miR-455 and PTEN 3′UTR predicted by TargetScan. **(B)** Dual-luciferase assays showing repression of wild-type PTEN-3′UTR following transfection of hsa-miR-455 mimics or a negative control. Data shown are the average of the three biological replicated experiments. The value obtained from first biological replica of co-transfection of negative control and luciferase reporter containing wild-type PTEN-3′UTR was designated as 1. **(C)** Potential binding sites between rno-miR-455 and PTEN 3′UTR predicted by RNAhybrid. **(D)** Dual-luciferase assays showing repression of wild-type PTEN-3′UTR following transfection of rno-miR-455 mimics or a negative control. Data shown are the average of the three biological replicated experiments. The value obtained from first biological replica of co-transfection of negative control and luciferase reporter containing wild-type PTEN-3′UTR was designated as 1. **(E)** Schematic illustration of luciferase reporter constructs for the miR-455 targeting site at the 3′UTR of PTEN. ^*^p < 0.05.

Potential binding sites between miR-455 and PTEN-3′UTR in both human and rat sequences were identified by RNA hybrid, Targetscan and miRbase (Figures 3A,C). A luciferase reporter system was constructed to investigate whether miR-455 regulate PTEN expression by targeting its 3′UTR. The PTEN-3′UTR flanking the entire predicted target sequence (wild type, WT) or the PTEN-3′UTR with a mutated target sequence (mutant type, MT) were cloned into a luciferase reporter vector, and co-transfected into 293T cells with either miR-455 or negative control (NC). Comparing to NC, co-transfection of has-miR-455 with the human WT PTEN 3′UTR sequence containing luciferase vector led to reduced luciferase activities; while this reduction was not seen when the co-transfection was performed with the MT PTEN 3′UTR sequence containing vector (Figure 3B). Similar findings were observed with the rat PTEN 3′UTR sequence and rno-miR-455, in which, the luciferase activities reduced when cells were co-transfected with rno-miR-455 and WT PTEN 3′UTR sequence containing vector but not MT 3′UTR sequence containing vector (Figure 3D). These findings suggested that miR-455 directly targeted the PTEN-3′UTR in regulating PTEN expression (Figure 3E).

### p65 Targets the miR-455-Promoter and Mediates p65 Induced Vascular Smooth Muscle Cell Proliferation and Phenotype Transformation

**Figure 4:**
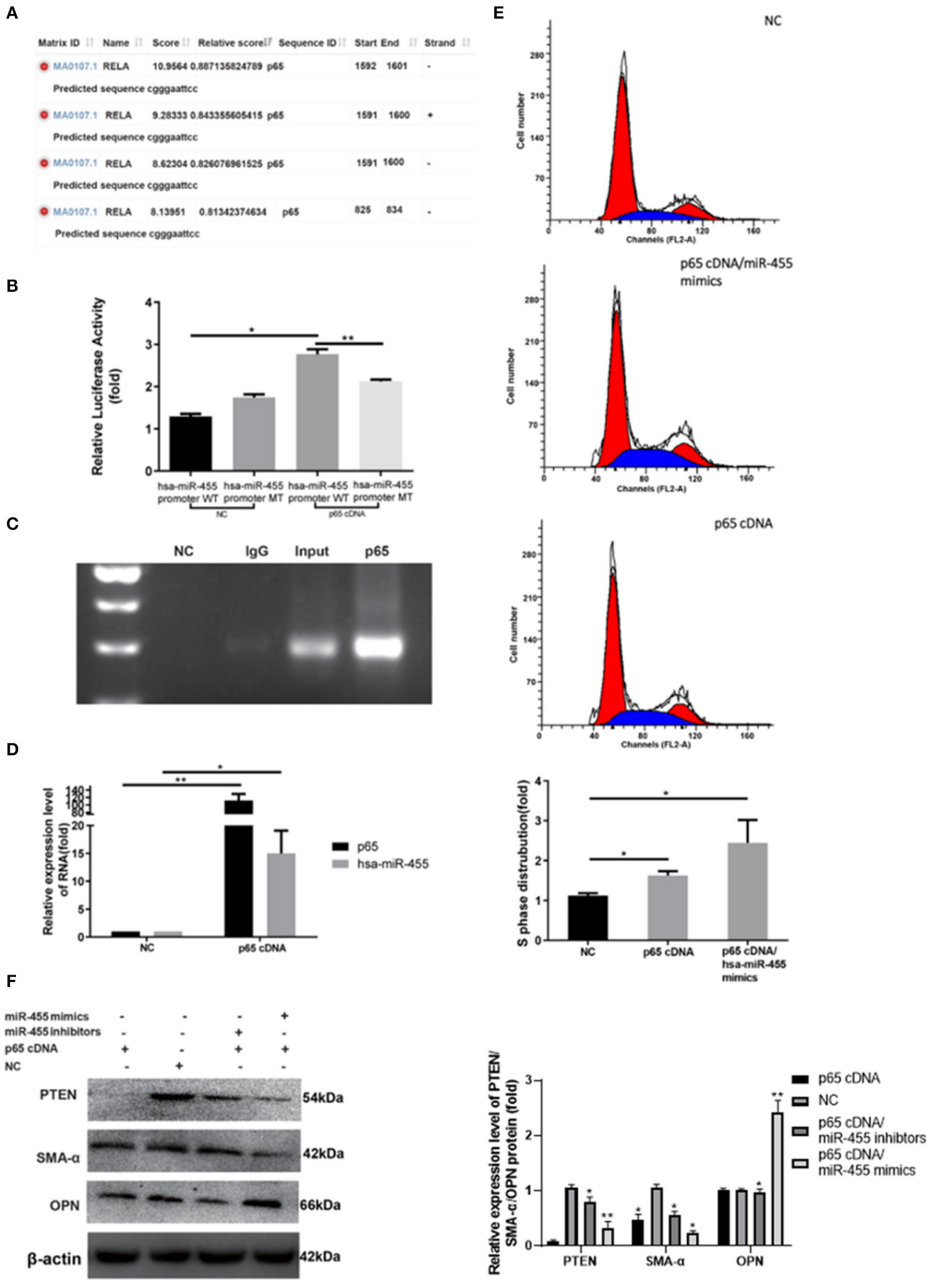
**(A)** Potential binding sites between p65 and precursor hsa-miR-455 promoter regions predicted by JASPAR. **(B)** Dual-luciferase assays showing p65 cDNA transfection upregulating luciferase activity with co-transfection of wild-type precursor hsa-miR-455 promoter region containing luciferase reporter vector, but not the mutant promoter region. Data shown are the average of the three biological replicated experiments. The value obtained from the first biological replica of co-transfection of negative control and luciferase reporter containing wild-type promoter region was designated as 1. **(C)** ChIP assays where p65 directly binds to the miR-455 promoter. **(D)** Expression levels of hsa-miR-455 or p65 in T/G HA-VSMCs transfected with p65 cDNA or a negative control examined by qRT-PCR. Hsa-miR-455 was normalized to U6 and p65 was normalized to β-actin. Data shown are the average of the three biological replicated experiments. The average value obtained from transfection of negative control was designated as 1. **(E)** Representative images of the cell cycle post-transfection with p65 cDNA or p65 cDNA/hsa-miR-455 (Blue region stands for S phase), quantification of the percentage of S phase cells was shown as the average of three biological replicated experiments. The percentage of S phase cells observed in the first negative control transfection experiment was designated as 1. **(F)** Representative images of Immunoblotting for PTEN/SMA-α/OPN expression levels in T/G HA-VSMCs after transfection. Three biological replicated experiments were performed. The values obtained from the negative control transfection in the first western blot experiment were designated as 1. ^*^p < 0.05, ^**^p < 0.01.

NF-κB p65/PTEN pathway has been implicated in VSMC remodeling in previous study. Several putative binding sites of p65 within has-miR-455 promoter region were identified with JASPAR (Figure 4A). The hsa-miR-455-promoter, which flanked the entire predicted target sequence (wild type, WT), or the hsa-miR-455-promoter containing a mutated target sequence (mutant type, MT) were cloned into the luciferase reporter vector. The resulting reporter vectors were co-transfected with p65 cDNA into 293T cells. As shown in Figure 4B, there were markable increased luciferase activities in cells transfected with WT vector along with p65 cDNA (Figure 4B). Some increased luciferase activities were observed when the cells were transfected with MT vector along with p65 cDNA, however, this increase was not statistically significant. Some unspecific binding and activation may explain the observation. Direct binding of p65 on has-miR-455 promoter was confirmed by ChIP assay where anti-p65 antibody was able to immunoprecipitated the promoter region of miR-455 (Figure 4C).

To confirm the role of p65 in miR-455 induced VSMC proliferation and phenotypic transformation, p65 cDNA was transfected into T/G HA-VSMC cells, with or without miR-455 co-transfection. qPCR analysis demonstrated that transfection of p65 cDNA resulted in p65 over-expression and upregulated hsa-miR-455 levels (Figure 4D). VSMCs transfected with p65 have increased S phase cell distribution, and this was further potentiated by co-transfected with miR-455 mimic (Figure 4E). p65 was able to induce VSMC phenotypic transformation. VSMC contractile marker SMA-α expression decreased upon p65 cDNA transfection, and it further decreased when cells were co-transfected with p65 cDNA and miR-455 mimic (Figure 4F). Co-transfection with miR-455 inhibitor, however, reversed the effect of p65 on SMA-α level. On the other hand, expression of OPN, VSMC synthetic marker, showed opposite pattern. The expression increased slightly upon p65 cDNA transfection, but greatly increased when cells were co-transfected with p65 and miR-455 mimic.

This phenotypic switching could be regulated through PTEN. Expression levels of PTEN were greatly decreased in cells transfected with p65 (Figure 4F).

### miR-455 Aggravates Vascular Stenosis *in vivo* and Regulates Vascular Smooth Muscle Cell Phenotype Transformation

**Figure 5:**
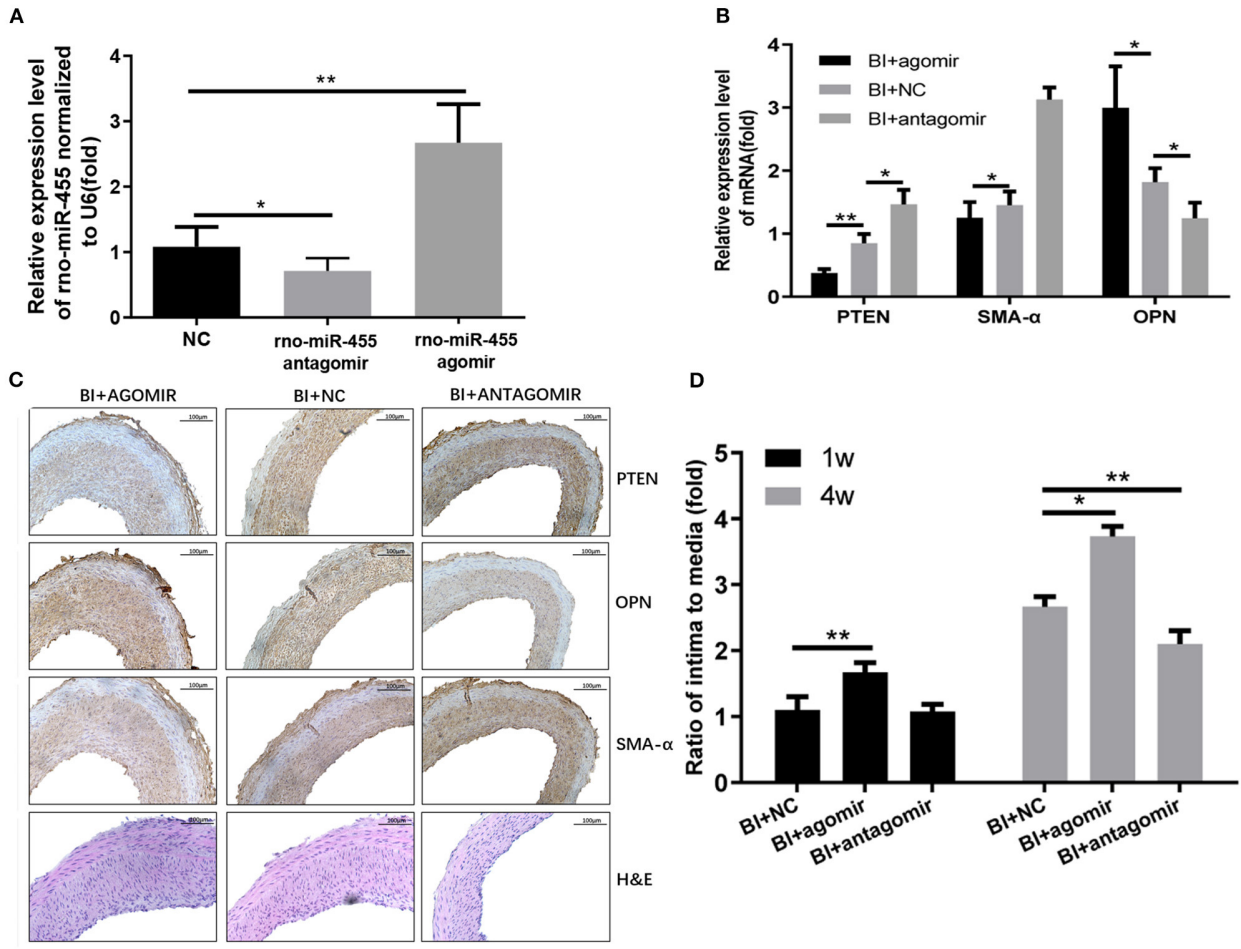
**(A)** Expression levels of rno-miR-455 in uninjured and balloon-injured (BI) arteries examined by qRT-PCR. Rno-miR-455 was normalized to U6. **(B)** Expression levels of PTEN, SMA-α and OPN in BI arteries transfected with a negative control, rno-miR-455 agomiR or rno-miR-455 antagomiR examined by qRT-PCR. mRNA was normalized to β-actin. The values obtained from the tissue samples in the group transfected with negative control were designated as 1. **(C)** Immunohistochemistry of PTEN, α-SMA and OPN in BI arteries transfected with a negative control, rno-miR-455 agomiR or rno-miR-455 antagomiR. **(D)** The degree of neointimal formation indicated by the ratio between intima and media, measured with Image-Pro Plus 6.0 software. The value obtained from the tissue sample of first animal transfected with negative control at week 1 time point was designated as 1. ^*^p < 0.05, ^**^p < 0.01.

The potential therapeutic effect of miR-455 was investigated in rat carotid artery balloon injury model. Rno-miR-455 agomiR or antagomiR was mixed with Matrigel, and transfected into carotid artery of the rats that underwent balloon injury procedure. All local transfections were completed following guidelines for a well-established balloon-injured (BI) artery rat model of restenosis. Twenty-four hours postoperative RT-qPCR confirmed that the transfection of agomiR increased the levels of miR-455 while antagomiR decreased the levels of miR-455 in carotid artery samples (Figure 5A). At 4 weeks post transfection, significantly decreased PTEN and SMA-α levels were detected in rno-miR-455 agomiR transfected artery samples, whereas at same time, OPN levels were significantly upregulated (Figures 5B,C). Transfection with agomiR increased neointimal formation at both 1 and 4 weeks after injury, as reflected by the increased ratio of intima to media ratio (*p* < 0.050), whereas antagomiR significantly reduced neointimal formation at 4 weeks after injury (*p* < 0.010) (Figure 5D).

## Discussion

Mature VSMCs are unique in that although they are quiescent normally in a contractile phenotype, they are not terminally differentiated, but present remarkable plasticity. The phenotypic switching from contractile to synthetic phenotype can be triggered by multiple environmental signals, including inflammation and injury. Once in the synthetic phenotype, VSMCs are capable of proliferation and migration, which may lead to atherosclerosis or stenosis.

A number of studies have explored the role of miRNAs in atherosclerosis and stenosis (30–32). Several circulating miRNAs are considered as biomarkers for restenosis (33).

Studies have shown that miR-455 is involved in multiple biological process. It is shown to be involved in modulating inflammation, a contributing factor in VSMC phenotypic switching, although its function varies depending on the system studied (18, 34, 35). miR-455/1281/191 have also been identified as potential biomarkers for abdominal aortic aneurysms (AAAs) (36).

In our study, we found miR-455 was over-expressed in both human stenotic artery samples and rat stenosis carotid artery tissues, which is consistent with work conducted by Choe et al. (22). The *in vitro* experiments with T/G HA-VSMC showed that proliferating cells expressed higher levels of miR-455, and this over-expression correlated with the decreased levels of PTEN and SMA-α, a VSMC contractile marker, and with the increased levels of VSMC synthetic marker OPN. The observation that miR-455 mimic transfection would induce cell proliferation and reduce the expression levels of PTEN and SMA-α, while miR-455 inhibitor transfection would have opposite effect, suggested that miR-455 may directly regulate the phenotypic transformation of VSMC.

PTEN, initially identified as a tumor suppressor gene (37–39), was later found to also play a role in the vascular pathological process, including inflammation, VSMC migration, proliferation and apoptosis (40–42). We identified potential target sequence of miR-455 in the PTEN 3′UTR in both human and rat genome, and were able to demonstrate that miR-455 modulated PTEN expression through the 3′UTR with luciferase report assays. Our study suggested that miR-455 may be involved in the regulation of phenotypic transformation of VSMC through modulating PTEN expression.

Using the well-established carotid artery balloon injury animal model, we showed miR-455 expressions were elevated after injury. When the injured tissue was transfected with miR-455 agomir, PTEN expression levels were repressed, and vascular remodeling was aggravated as reflecting by intima to media ratio. However, when the injured tissue was transfected with miR-455 antagomir, tissue PTEN levels were increased, and vascular remodeling was relieved at 4 weeks post injury and transfection.

NF-κB is critical to multiple biological functions. As a member of the NF-κB family, p65 participates in many biological processes, including endothelial inflammatory responses, retinal endothelial cells damage (43) and vascular endothelial cell apoptosis in limb ischemia/reperfusion injuries (44). P65 participates in diabetic mouse carotid artery stenosis (45) and regulates VSMC phenotypes (46). VSMC inflammation was shown to be related to p65 (47) and p65 regulates cholesterol efflux to HDL from VSMCs (48).

We identified several potential binding sites of p65 in miR-455 promoter region and assessed their validity. We demonstrated that p65 can directly bind to miR455 promoter region, and modulate the luciferase activity through miR-455 promoter region containing potential binding sites. Furthermore, we found that over-expression of p65 increased the expression of miR-455 and promote VSMC proliferation, this effect could be further potentiated by over-expression of miR-455, suggesting that p65 was able to induce VSMC phenotypic transformation via miR-455/PTEN pathway.

Overall, our findings indicated that miR-455 plays a key role in the dynamics of vascular disease progression. The p65/miR-455/PTEN pathway may be an attractive therapeutic target for atherosclerosis or vascular restenosis. Our findings that antagomir-455 was effective in delaying vascular restenosis after balloon injury *in vivo*, suggested that it is possible to inhibit VSMC phenotypic transformation via intervention.

Further analysis for the mechanisms underlying the dynamics influencing miR-455 is needed, especially in the context of VSMCs and restenosis with transgenic animal models. However, our findings provide direct evidence for the important function of miR-455 in VSMC remodeling, which is possible to be a potential target for anti-stenosis therapy.

## Data Availability Statement

The original contributions presented in the study are included in the article/Supplementary Materials, further inquiries can be directed to the corresponding author/s.

## Ethics Statement

The studies involving human participants were reviewed and approved by Ethics Committee of the First Affiliated Hospital of China Medical University. The patients/participants provided their written informed consent to participate in this study. The animal study was reviewed and approved by Ethics Committee of China Medical University.

## Author Contributions

RL: conceptualization, methodology, data curation, and writing—original draft preparation. JL: methodology and software. LW: software and validation. XL and JZ: visualization and investigation. WS: writing—reviewing and editing. XH: Investigation. SX: supervision. All authors contributed to the article and approved the submitted version.

## Conflict of Interest

The authors declare that the research was conducted in the absence of any commercial or financial relationships that could be construed as a potential conflict of interest.

## References

[1] Meyer A Schinz K Lang W Schmid A Regus S Rother U. Outcomes and influence of the pedal arch in below-the-knee angioplasty in patients with end-stage renal disease and critical limb ischemia. Ann Vasc Surg. (2016) 35:121–9. 10.1016/j.avsg.2016.01.039 27238998

[2] Huang SC Wang M Wu WB Wang R Cui J Li W. miR-22-3p inhibits arterial smooth muscle cell proliferation and migration and neointimal hyperplasia by targeting HMGB1 in arteriosclerosis obliterans. Cell Physiol Biochem. (2017) 42:2492–506. 10.1159/000480212 28848136

[3] Lee R Feinbaum R Ambros V. A short history of a short RNA. Cell. (2004) 116:S89–92, 1–96. 10.1016/S0092-8674(04)00035-2 15055592

[4] Filipowicz W Bhattacharyya SN Sonenberg N. Mechanisms of post-transcriptional regulation by microRNAs: are the answers in sight? Nat Rev Genet. (2008) 9:102–14. 10.1038/nrg2290 18197166

[5] Ha M Kim VN. Regulation of microRNA biogenesis. Nat Rev Mol Cell Biol. (2014) 15:509–24. 10.1038/nrm3838 25027649

[6] Urbich C Kuehbacher A Dimmeler S. Role of microRNAs in vascular diseases, inflammation, and angiogenesis. Cardiovasc Res. (2008) 79:581–8. 10.1093/cvr/cvn156 18550634

[7] Liu S Yang Y Jiang S Tang N Tian J Ponnusamy M. Understanding the role of non-coding RNA. (ncRNA) in stent restenosis. Atherosclerosis. (2018) 272:153–61. 10.1016/j.atherosclerosis.2018.03.036 29609130

[8] Lv J Wang L Zhang J Lin R Wang L Sun W. Long noncoding RNA H19-derived miR-675 aggravates restenosis by targeting PTEN. Biochem Biophys Res Commun. (2018) 497:1154–61. 10.1016/j.bbrc.2017.01.011 28063931

[9] Santulli G Wronska A Uryu K Diacovo TG Gao M Marx SO. A selective microRNA-based strategy inhibits restenosis while preserving endothelial function. J Clin Invest. (2014) 124:4102–14. 10.1172/JCI76069 25133430 PMC4153706

[10] McDonald RA Halliday CA Miller AM Diver LA Dakin RS Montgomery J. Reducing in-stent restenosis: therapeutic manipulation of miRNA in vascular remodeling and inflammation. J Am Coll Cardiol. (2015) 65:2314–27. 10.1016/j.jacc.2015.03.549 26022821 PMC4444526

[11] Guan Y Cai B Wu X Peng S Gan L Huang D. microRNA-352 regulates collateral vessel growth induced by elevated fluid shear stress in the rat hind limb. Sci Rep. (2017) 7:6643. 10.1038/s41598-017-06910-9 28751690 PMC5532297

[12] Yunqi H Fangrui Y Yongyan Y Yunjian J Wenhui Z Kun C. miR-455 Functions as a tumor suppressor through targeting GATA6 in colorectal cancer. Oncol Res. (2019) 27:311–6. 10.3727/096504018X15220579006875 29615149 PMC7848416

[13] Chai L Kang XJ Sun ZZ Zeng MF Yu SR Ding Y. miR-497-5p, miR-195-5p and miR-455-3p function as tumor suppressors by targeting hTERT in melanoma A375 cells. Cancer Manag Res. (2018) 10:989–1003. 10.2147/CMAR.S163335 29760567 PMC5937487

[14] Sun Y Wang Y Yang H Xu Y Yu H. miR-455-3p functions as a tumor suppressor in colorectal cancer and inhibits cell proliferation by targeting TPT1. Int J Clin Exp Pathol. (2018) 11:2522–9. 31938365 PMC6958251

[15] Li Z Meng Q Pan A Wu X Cui J Wang Y. MicroRNA-455-3p promotes invasion and migration in triple negative breast cancer by targeting tumor suppressor EI24. Oncotarget. (2017) 8:19455–66. 10.18632/oncotarget.14307 28038450 PMC5386697

[16] Ni X Ding Y Yuan H Shao J Yan Y Guo R. Long non-coding RNA ZEB1-AS1 promotes colon adenocarcinoma malignant progression via miR-455-3p/PAK2 axis. Cell Prolif . (2020) 53:e12723. 10.1111/cpr.12723 31828845 PMC6985675

[17] Singh AK Rooge SB Varshney A Vasudevan M Bhardwaj A Venugopal SK. Global microRNA expression profiling in the liver biopsies of hepatitis B virus-infected patients suggests specific microRNA signatures for viral persistence and hepatocellular injury. Hepatology. (2018) 67:1695–709. 10.1002/hep.29690 29194684

[18] Cheng F Hu H Sun K Yan F Geng Y. miR-455-3p enhances chondrocytes apoptosis and inflammation by targeting COL2A1 in the *in vitro* osteoarthritis model. Biosci Biotechnol Biochem. (2020) 84:695–702. 10.1080/09168451.2019.1690974 31809639

[19] Fiorillo AA Tully CB Damsker JM Nagaraju K Hoffman EP Heier CR. Muscle miRNAome shows suppression of chronic inflammatory miRNAs with both prednisone and vamorolone. Physiol Genomics. (2018) 50:735–45. 10.1152/physiolgenomics.00134.2017 29883261 PMC6172612

[20] Torabi S Tamaddon M Asadolahi M Shokri G Tavakoli R Tasharrofi N. miR-455-5p downregulation promotes inflammation pathways in the relapse phase of relapsing-remitting multiple sclerosis disease. Immunogenetics. (2019) 71:87–95. 10.1007/s00251-018-1087-x 30310937

[21] Zhou C Chen Y Kang W Lv H Fang Z Yan F. miR-455-3p-1 represses FGF7 expression to inhibit pulmonary arterial hypertension through inhibiting the RAS/ERK signaling pathway. J Mol Cell Cardiol. (2019) 130:23–35. 10.1016/j.yjmcc.2019.03.002 30858037

[22] Choe N Kwon JS Kim JR Eom GH Kim Y Nam KI. The microRNA miR-132 targets Lrrfip1 to block vascular smooth muscle cell proliferation and neointimal hyperplasia. Atherosclerosis. (2013) 229:348–55. 10.1016/j.atherosclerosis.2013.05.009 23880186

[23] Sedding DG Widmer-Teske R Mueller A Stieger P Daniel JM Gunduz D. Role of the phosphatase PTEN in early vascular remodeling. PLoS ONE. (2013) 8:e55445. 10.1371/journal.pone.0055445 23533567 PMC3606387

[24] Lu S Strand KA Mutryn MF Tucker RM Jolly AJ Furgeson SB. PTEN (Phosphatase and Tensin Homolog) protects against ang II (Angiotensin II)-induced pathological vascular fibrosis and remodeling-brief report. Arterioscler Thromb Vasc Biol. (2020) 40:394–403. 10.1161/ATVBAHA.119.313757 31852223 PMC7059862

[25] Moulton KS Li M Strand K Burgett S McClatchey P Tucker R. PTEN deficiency promotes pathological vascular remodeling of human coronary arteries. JCI Insight. (2018) 3:e97228. 10.1172/jci.insight.97228 29467331 PMC5916252

[26] Furgeson SB Simpson PA Park I Vanputten V Horita H Kontos CD. Inactivation of the tumour suppressor, PTEN, in smooth muscle promotes a pro-inflammatory phenotype and enhances neointima formation. Cardiovasc Res. (2010) 86:274–82. 10.1093/cvr/cvp425 20051384 PMC2856191

[27] Tulis DA. Rat carotid artery balloon injury model. Methods Mol Med. (2007) 139:1–30. 10.1007/978-1-59745-571-8_1 18287662 PMC2819386

[28] Stepien O Gogusev J Zhu DL Iouzalen L Herembert T Drueke TB. Amlodipine inhibition of serum-, thrombin-, or fibroblast growth factor-induced vascular smooth-muscle cell proliferation. J Cardiovasc Pharmacol. (1998) 31:786–93. 10.1097/00005344-199805000-00019 9593080

[29] Wen X Li H Sun H Zeng A Lin R Zhao J. miR-455-3p reduces apoptosis and alleviates degeneration of chondrocyte through regulating PI3K/AKT pathway. Life Sci. (2020) 253:117718. 10.1016/j.lfs.2020.117718 32343998

[30] Gareri C De Rosa S Indolfi C. MicroRNAs for restenosis and thrombosis after vascular injury. Circ Res. (2016) 118:1170–84. 10.1161/CIRCRESAHA.115.308237 27034278

[31] Chen LJ Lim SH Yeh YT Lien SC Chiu JJ. Roles of microRNAs in atherosclerosis and restenosis. J Biomed Sci. (2012) 19:79. 10.1186/1423-0127-19-79 22931291 PMC3438039

[32] Yamakuchi M. MicroRNAs in vascular biology. Int J Vasc Med. (2012) 2012:794898. 10.1155/2012/794898 23056947 PMC3463915

[33] Varela N Lanas F Salazar LA Zambrano T. The current state of MicroRNAs as restenosis biomarkers. Front Genet. (2019) 10:1247. 10.3389/fgene.2019.01247 31998354 PMC6967329

[34] Shao M Xu Q Wu Z Chen Y Shu Y Cao X. Exosomes derived from human umbilical cord mesenchymal stem cells ameliorate IL-6-induced acute liver injury through miR-455-3p. Stem Cell Res Ther. (2020) 11:37. 10.1186/s13287-020-1550-0 31973730 PMC6979401

[35] Gu Q Wang B Zhao H Wang W Wang P Deng Y. LncRNA promoted inflammatory response in ischemic heart failure through regulation of miR-455-3p/TRAF6 axis. Inflamm Res. (2020) 69:667–81. 10.1007/s00011-020-01348-8 32350569

[36] Zhang W Shang T Huang C Yu T Liu C Qiao T. Plasma microRNAs serve as potential biomarkers for abdominal aortic aneurysm. Clin Biochem. (2015) 48:988–92. 10.1016/j.clinbiochem.2015.04.016 25916817

[37] Wise HM Hermida MA Leslie RN. Prostate cancer, PI3K, PTEN and prognosis. Clin Sci. (2017) 131:197–210. 10.1042/CS20160026 28057891

[38] Kechagioglou P Papi RM Provatopoulou X Kalogera E Papadimitriou E Grigoropoulos P. Tumor suppressor PTEN in breast cancer: heterozygosity, mutations and protein expression. Anticancer Res. (2014) 34:1387–400. 24596386

[39] Lee MS Jeong MH Lee HW Han HJ Ko A Hewitt SM. PI3K/AKT activation induces PTEN ubiquitination and destabilization accelerating tumourigenesis. Nat Commun. (2015) 6:7769. 10.1038/ncomms8769 26183061 PMC4518267

[40] Zhang L Zhou C Qin Q Liu Z Li P. LncRNA LEF1-AS1 regulates the migration and proliferation of vascular smooth muscle cells by targeting miR-544a/PTEN axis. J Cell Biochem. (2019) 120:14670–8. 10.1002/jcb.28728 31016789

[41] Wang M Liu M Ni T Liu Q. miR214 mediates vascular inflammation and apoptosis via PTEN expression. Mol Med Rep. (2018) 18:2229–36. 10.3892/mmr.2018.9185 29916551

[42] Wang S Cheng Z Chen X. Promotion of PTEN on apoptosis through PI3K/Akt signal in vascular smooth muscle cells of mice model of coronary heart disease. J Cell Biochem. (2019) 120:14636–44. 10.1002/jcb.28725 31090947

[43] Song Y Tian X Wang X Feng H. Vascular protection of salicin on IL-1beta-induced endothelial inflammatory response and damages in retinal endothelial cells. Artif Cells Nanomed Biotechnol. (2019) 47:1995–2002. 10.1080/21691401.2019.1608220 31106593

[44] Mi L Zhang Y Xu Y Zheng X Zhang X Wang Z. HMGB1/RAGE pro-inflammatory axis promotes vascular endothelial cell apoptosis in limb ischemia/reperfusion injury. Biomed Pharmacother. (2019) 116:109005. 10.1016/j.biopha.2019.109005 31136947

[45] Ji Y Ge Y Xu X Ye S Fan Y Zhang J. Vildagliptin reduces stenosis of injured carotid artery in diabetic mouse through inhibiting vascular smooth muscle cell proliferation via ER Stress/NF-kappaB pathway. Front Pharmacol. (2019) 10:142. 10.3389/fphar.2019.00142 30858802 PMC6397934

[46] Kong P Yu Y Wang L Dou YQ Zhang XH Cui Y. circ-Sirt1 controls NF-kappaB activation via sequence-specific interaction and enhancement of SIRT1 expression by binding to miR-132/212 in vascular smooth muscle cells. Nucleic Acids Res. (2019) 47:3580–93. 10.1093/nar/gkz141 30820544 PMC6468289

[47] Qiu L Xu C Chen J. Downregulation of the transcriptional co-activator PCAF inhibits the proliferation and migration of vascular smooth muscle cells and attenuates NF-kappaB-mediated inflammatory responses, Biochem Biophys Res Commun, (2019) 513:41–8. 10.1016/j.bbrc.2019.03.157 30935684

[48] Cai C Zhu H Ning X Li L Yang B Chen S. LncRNA ENST00000602558.1 regulates ABCG1 expression and cholesterol efflux from vascular smooth muscle cells through a p65-dependent pathway. Atherosclerosis. (2019) 285:31–9. 10.1016/j.atherosclerosis.2019.04.204 31003090

