# Supplementary figures and images for: Potential Target miR-455 Delaying Arterial Stenosis Progression Through PTEN
Source: Front Cardiovasc Med. 2021 Feb 23;8:611116. doi: 10.3389/fcvm.2021.611116 (PMC7940831; doi:10.3389/fcvm.2021.611116)

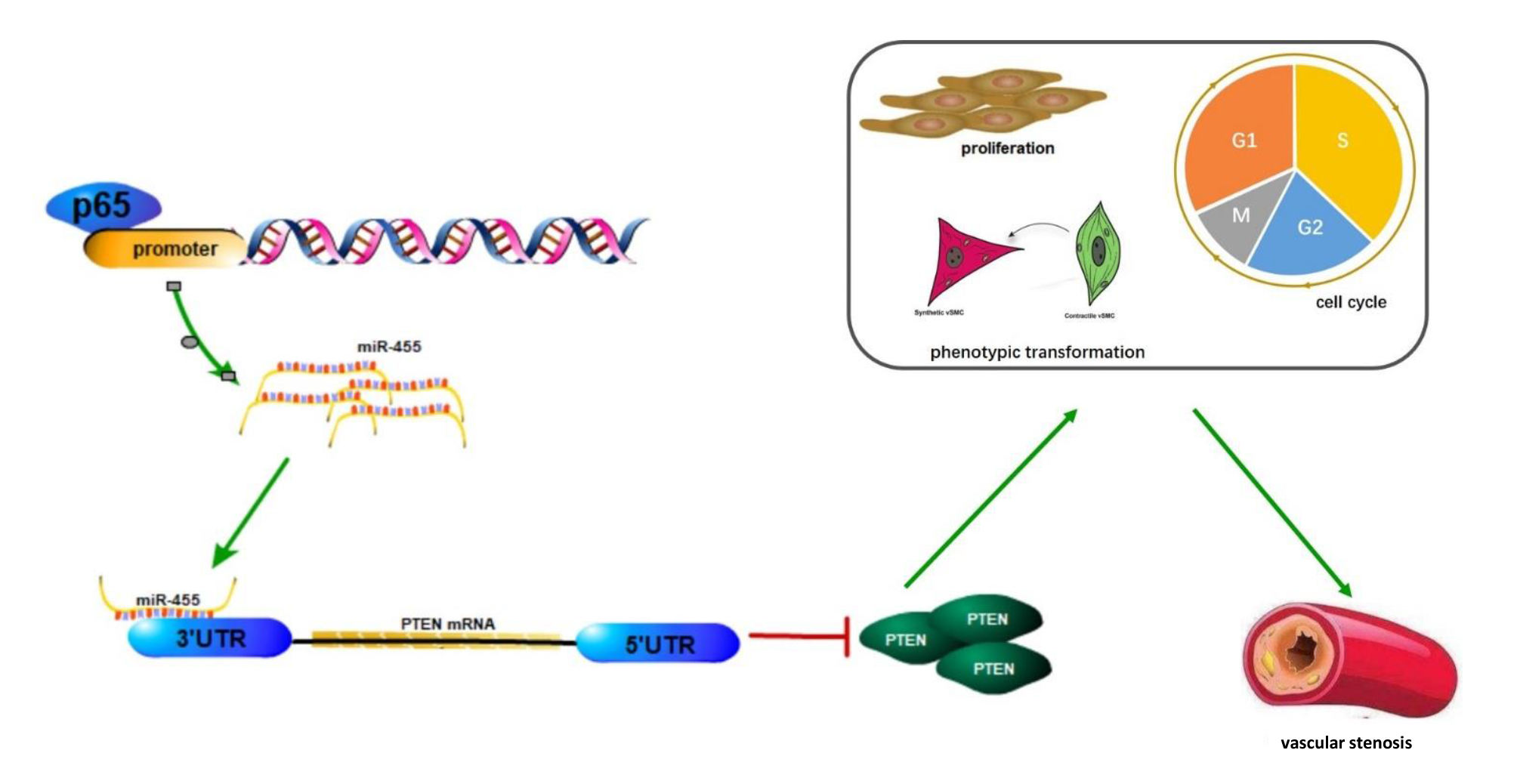

Supplement: Supplementary file 1 [file Image_1.TIF]
